# Is acupuncture safe in the ICU? A systematic review and meta-analysis

**DOI:** 10.3389/fmed.2023.1190635

**Published:** 2023-08-24

**Authors:** Eyal Ben-Arie, Bernice Jeanne Lottering, Fang-Pey Chen, Wen-Chao Ho, Yu-Chen Lee, Chanya Inprasit, Pei-Yu Kao

**Affiliations:** ^1^Graduate School of Acupuncture Science, China Medical University, Taichung, Taiwan; ^2^Institute of Traditional Medicine, School of Medicine, National Yang Ming Chiao Tung University, Taipei, Taiwan; ^3^Department of Public Health, China Medical University, Taichung, Taiwan; ^4^Department of Acupuncture, China Medical University Hospital, Taichung, Taiwan; ^5^Chinese Medicine Research Center, China Medical University, Taichung, Taiwan; ^6^Suphanburi Campus Establishment Project, Kasetsart University, Suphan Buri, Thailand; ^7^Surgical Intensive Care Unit, China Medical University Hospital, Taichung, Taiwan; ^8^Division of Thoracic Surgery, Department of Surgery, China Medical University Hospital, Taichung, Taiwan

**Keywords:** acupuncture, intensive care unit, safety, adverse events, adverse effects

## Abstract

**Background and purpose:**

The safety of interventions for critically ill patients is a crucial issue. In recent years, several studies have treated critically ill patients with acupuncture. However, the safety of acupuncture in this setting remains to be systematically measured.

**Methods:**

In May 2022, the electronic databases of PubMed and the Cochrane Library were searched for studies comparing acupuncture interventions to control interventions in critically ill patients. Study outcomes examined the incidence of severe adverse events (AEs), minor AEs, adverse reactions, ICU stays, and 28-day mortality.

**Results:**

A total of 31 articles were analyzed, and no serious AEs related to acupuncture treatment were identified. No significant differences were found between the groups in the meta-analysis of minor AEs (risk ratio [RR] 5.69 [0.34, 96.60], *P* = 0.23, *I*^2^ = 76%). A reduced risk in the incidence of adverse reactions following acupuncture intervention was evidenced (RR 0.33 [0.22, 0.50], *P* = 0.00001, *I*^2^ = 44%). The patients in the acupuncture arm spent significantly less time in the intensive care unit (ICU) (Mean difference −1.45 [−11.94, −10.97], *P* = 0.00001, *I*^2^ = 56%) and also exhibited lower 28-day mortality rates (odds ratio 0.61 [0.48, 0.78], *P* = 0.0001, *I*^2^ = 0%).

**Conclusion:**

There is no evidence to indicate a higher risk of severe or minor AEs in patients who receive acupuncture. Acupuncture demonstrated favorable results in both ICU stay and 28-day mortality measurements, in addition to presenting with fewer adverse reactions compared to routine ICU care. However, the low certainty of the evidence resulting from a high risk of bias in the included studies merits substantial consideration, and further research is still warranted.

**Systematic review registration:**

https://www.crd.york.ac.uk/prospero/display_record.php?RecordID=142131, identifier: CRD42020142131.

## Introduction

Critically ill patients are identified to be at high risk for developing adverse events (AEs) from conventional intensive care unit (ICU) treatments, as demonstrated by the affiliated incidence of 25.4 per 100 critically ill hospital admissions. The most commonly reported AEs that occur in this setting are respiratory complications, hospital-acquired infections, organ failures, surgical complications, hemorrhage, and gastrointestinal complications, among various others (1). AEs that occur in critically ill patients are most commonly associated with the administration of certain medications and may also be attributed to serious errors committed by the medical staff, although an array of influencing factors may be involved in the incidence of AEs. Drug-related AEs are one of the most prevalent causes and are also among the most well-studied in the current literature. Furthermore, a majority of ICU interventions include the implementation of extracorporeal membrane oxygenation, which is known to induce a certain percentage of complications (2–4).

The efficacy of acupuncture treatment has been solidly established within clinical practice, and acupuncture safety is globally recognized, with its usage increasing and expanding in recent decades (5). Acupuncture is rarely used in critical care settings, primarily due to the uncertainty in its' affiliated safety index in ICU patients. Despite this, a plethora of evidence exists to validate the safety of this treatment, including the therapeutic efficacy being confirmed as well as the occurrence of serious AEs being extremely rare in the overall population (6). A retrospective study investigating the incidence of acupuncture AEs in a total of 73,406 patients with low back or neck pain found that around 7% will suffer from AEs, of which < 2% required treatment for those AEs, and only 0.03% required treatment in the hospital for those AEs (7). Another retrospective study including 229,230 patients receiving an average of 10 acupuncture treatments for various ailments found that an average of 8% of patients will experience AEs, and around 2% will require treatments for those AEs. Furthermore, local site bleeding, or hematoma, was the most commonly reported AE incidence (8). Although rare, the primary severe AEs reported from acupuncture treatment are related to mycobacterium infections, organ and tissue injuries, pneumothorax, nerve injury, and heart injury (9). Proper, professional administration of acupuncture treatment is imperative to avoid a multitude of AEs and includes the application of sterile needles, skin disinfection, avoidance of dangerous acupoints, proper needling depth, and attention to the physical attributes of individual patients to allow for effective prevention of most, if not all, of the aforementioned AEs. In a systematic review conducted by Formenti et al. (10) the potential effectiveness and applicability of acupuncture for critically ill patients were explored. However, to the best of our knowledge, no previous research has been conducted to investigate the safety of acupuncture in critically ill patients through a systematic review and meta-analysis.

The aim of this study was to provide a detailed analysis regarding the safety of acupuncture-related treatment in critically ill patients within an intensive care setting across a cohort over 18 years old, including any race, gender, or ICU affliction. The specific intervention will include acupuncture, electro-acupuncture (EA), auricular therapy, or acupoint stimulations (electric or manual), with or without the use of other medications. The control treatment may include any treatment. Outcomes were presented according to the incidence of severe AEs, minor AEs, adverse reactions, ICU stays, and mortality.

## Methods

This systematic review was registered on the Prospective Register of Ongoing Systematic Reviews (PROSPERO) with registration No. CRD42020142131 on 28 April 2020. This work followed the Preferred Reporting Items for Systematic Reviews and Meta-Analyses (PRISMA) harm checklist recommendations (11).

### Eligibility criteria

#### Types of studies

Case reports, retrospective studies, and clinical trials in any form (randomized and non-randomized, pilot) were considered for inclusion as long as they were conducted in an ICU and involved acupuncture-related treatment.

#### Participants/population

We included critically ill patients submitted to a medical or surgical intensive care department who were over 18 years old without any restriction regarding gender or race.

#### Interventions/exposure

Interventions included acupuncture, EA, auricular therapy, and acupoint stimulations (electric or manual, but excluding manual acupressure) with or without additional treatments. The commencement of the intervention was required after the admission to the ICU or continued throughout the ICU period.

Control intervention was determined to include any type of intervention, including control acupuncture, sham acupuncture, specific medication administration, and routine ICU care.

#### Outcome measurement

Our primary outcome is evaluating the incidence of severe AEs (including infections, organ and tissue injuries, pneumothorax, nerve and heart injury, as well as mortality) in relation to acupuncture, EA, auricular therapy, and acupoint stimulations.

For secondary outcomes, minor AEs, adverse reactions, and ICU stays will be analyzed, in addition to combined mortality (including ICU, hospital, and 28-day mortality) alongside the specifically reported 28-day mortality rate. Minor AEs are defined as AEs that are inclusive of subcutaneous hematoma, minor hemorrhage, serious pain, fainting, and local infections; other AEs that do not fit the category of severe AEs or adverse reactions; or those described as minor AEs in the included individual studies. An adverse reaction was defined as a deterioration in the patient's condition that occurred in close proximity to the intervention, yet its association with the intervention was not clear, in addition to individual cases that the included studies categorized as such.

### Literature search

Regarding the literature search and inclusion criteria, the electronic databases of both PubMed and the Cochrane library were thoroughly searched without restrictions. The keywords were a combination of the following: “acupuncture OR electro-acupuncture OR auricular therapy OR acupoints” AND “critically ill OR intensive care OR ICU” AND “safe OR adverse event OR adverse effect.” The search was conducted in May 2022. The reference lists of studies were further examined for other relevant studies. Animal studies were excluded. Due to language restrictions, only English and Chinese language studies were included.

### Data selection

Two independent researchers (EBA and CI) reviewed the study's titles and abstracts. When relevant, full-text articles were examined for final inclusion. The study section sheet was completed for each study according to the established inclusion criteria, with rationales provided for exclusion. Study selection information was presented in accordance with the PRISMA harm flowchart. All studies involving acupuncture intervention in the ICU were included, with the reporting of AEs being reviewed in each study. Studies that did not mention any AEs were still included for measuring the overall safety of the intervention. Any disagreements in regard to study selection were resolved by discussion with a third author.

### Data extraction and management

Data from the included studies were extracted by three independent researchers (EBA, CI, and KPY), where two researchers focused on English language studies (EBA and CI), and one researcher focused on Chinese language studies (KPY). The researchers completed a data extraction sheet that includes the following information: first author, published year, study type, study location, age, gender, ethnicity, patient condition, sample size, randomization, allocation, blinding information, acupuncture and drug dosage, treatment frequency, treatment duration, treatment follow-up time, acupuncturist years of experience, and education level. With regard to AE-related details, the following information was included: number of patients suffering from AEs, type of AEs, acupoints selected, patient recovery outcome, follow-up time, number of events per patient, patient risk factors before acupuncture, total hospital stay in days with and without AEs, and total mortality. Upon the completion of the data extraction sheets, any disagreements were resolved through discussion.

### Missing data

With regard to missing data, the original published data were included as reported in the original studies. The implications of missing data cases were extrapolated in the discussion section of this manuscript.

### Risk of bias assessment

The Cochrane collaboration's tool for assessing the risk of bias in randomized trials was employed to assess this aspect accordingly (12).

### Meta-bias(es)

Publication bias and outcome reporting bias were addressed by a search of clinicalTrials.gov. Unpublished acupuncture studies that were conducted in an ICU setting were excluded from this systematic review and meta-analysis but were discussed further in the results section.

### Data synthesis

Meta-analyses were performed according to the following measurements: minor AEs following acupuncture, adverse reactions, a combination of AEs or adverse reactions, ICU stay, and overall safety of the intervention (including zero events). Meta-analyses were also conducted on the 28-day mortality and combined mortality (with respect to both hospital and 28-day mortality follow-ups).

For statistical analysis, the Review Manager Version 5.4 (Copenhagen: The Nordic Cochrane Center, The Cochrane Collaboration, 2014) was used to conduct all related meta-analyses. Dichotomous outcomes with 95% confidence intervals (CIs) were recorded. Heterogeneity tests were conducted using the χ^2^ test and *I*^2^ test. An *I*^2^ score of 50% or greater, or a significant χ^2^ test, indicates the existing heterogeneity. In case of high heterogeneity, a random-effects model was used. In instances where existing heterogeneity was not found, a fixed-effect model was used. In addition to the meta-analysis, a narrative description of the related study results was provided. The risk ratio was used for analyses of minor AEs, severe AEs, or adverse reactions. The risk difference was employed to describe the overall safety analysis, inclusive of zero AEs. The mean difference was used in the ICU days analysis, and the odds ratio was used to depict the mortality analyses.

For publication bias, funnel plots were visually analyzed, and the Egger's regression test for a 2-tailed *p*-value was additionally utilized to analyze for small study effects as determined through the Comprehensive Meta-Analysis V4 software.

### Subgroups and sensitivity analyses

For subgroup analyses, the studies were divided by the type of intervention used: Pyonex/Epidermal tacks acupuncture, MA, MA with herbal medicine, EA, EA with herbal medicine, TENS, or acupoint catgut embedding.

Sensitivity analyses were conducted by systematically omitting one of the trials.

### Confidence in cumulative estimate

The Grading of Recommendations Assessment, Development, and Evaluation (GRADE) method was used for measuring the quality of evidence. Risk of bias, consistency, directness, precision, and publication bias were employed for grading our confidence in the evidence. Accordingly, the quality of the evidence was divided into four grades: high, moderate, low, and very low. A GRADE summary of findings table was generated by the “GRADEpro” website: https://www.gradepro.org/.

## Results

**Figure 1:**
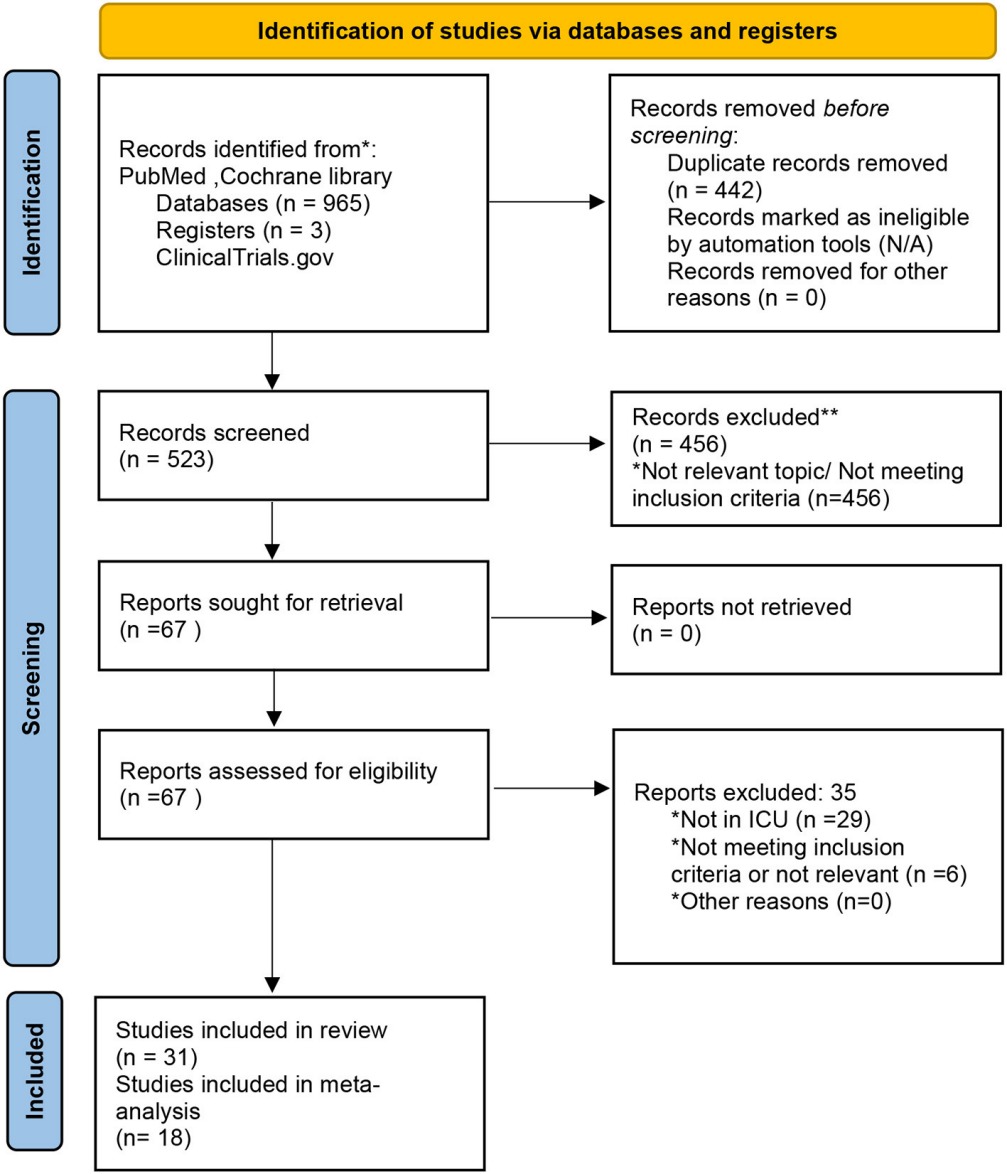
Study Flow chart depicting the identification, screening, eligibility, and inclusion process of the various studies incorporated in the systematic review and meta-analysis. Adapted from Page et al. (13), licensed under CC-BY-4.0.

An electronic database search on publications without time or study type search restrictions revealed a total of 965 publications. After removing duplications, a total of 523 publications remained. After the screening of titles and abstracts, there were 456 articles that did not meet the inclusion criteria or were not related to this topic, and 29 articles that were not inclusive of an ICU setting. Finally, a total of 31 articles were analyzed and synthesized for a systematic review, and 18 studies were included in the meta-analysis (Figure 1).

The general information for the 2,947 patients included in the 31 articles is displayed in Table 1. A majority of the original publications in this specific field originated in China (23 papers), followed by Iran (2 studies), while other countries reported 1 article each. A randomized control trial was the most popular study design (26 articles). However, an appropriate blinding technique for acupuncture investigation, known as the double-blind scheme, was utilized by only one published paper [AminiSaman et al. (25)]. Randomized control single-blind trials including patient blinding were mentioned in three studies [Ben-Arie et al. (16), Zhang et al. (18), Xing et al. (15)].

**Table 1:** The table describes details of the study design in the included studies.

**Study**	**Year**	**Country**	**N**	**Study type**	**Randomized technique**	**Blinding**
Wang et al. (14)	2022	China	88	Randomized control trial	-	No
Xing et al. (15)	2022	China	500	Multicenter single-blind randomized controlled trial	Random number table	Patients
Ben-Arie et al. (16)	2021	Taiwan	26	Single-blind randomized control trial	Computer-based simple random sampling	Patients
Xing et al. (17)	2021	China	100	Randomized control trial	Table of random numbers	No
Zhang et al. (18)	2021	China	92	Single-blind randomized control trial	Computer-generated numbers	Patients
Liu et al. (19)	2020	China	100	Randomized control trial	-	No
Liu et al. (20)	2020	China	49	Multi-center randomized control trial	-	No
Wang et al. (21)	2020	China	55	Randomized control trial	Random number table	No
Zheng et al. (22)	2020	China	70	Randomized control trial	Random number table	No
Li et al. (23)	2019	China	40	Randomized control trial	SPSS software random sampling	No
Liu et al. (24)	2019	China	49	Randomized control trial	Table of random numbers	No
AminiSaman et al. (25)	2018	Iran	40	Randomized control trial	Even and odd numbers random sampling	Double-blind
Meng et al. (26)	2018	China	82	Randomized control trial	SPSS software random sampling	Statistical researcher
Meng et al. (27)	2018	China	71	Randomized control trial	Random number table	Researcher
Bashar et al. (28)	2017	Iran	60	Randomized control trial	Computer generated block-randomized	Patients
Bataille et al. (29)	2017	France	10	Non-randomized no controlled study	No	No
Feeney et al. (30)	2017	USA	576	Non-randomized feasibility study	-	No
Matsumoto-Miyazaki et al. (31)	2017	Japan	59	Non-randomized observational study	-	No
Wang et al. (32)	2017	China	296	Multicenter, prospective, observational, non-randomized cohort study	-	No
Yang et al. (33)	2016	China	58	Randomized controlled trial	Block randomization.	No
Wang et al. (34)	2015	China	56	Randomized control trial	Random number table	No
Xiao et al. (35)	2015	China	90	Randomized control trial	Random number table	No
Yu et al. (36)	2015	China	20	Randomized pilot study	Random number table	No
Xiao et al. (37)	2014	China	60	Randomized control trial	Random number table	No
Zheng et al. (38)	2012	China	45	Exploratory study	Sealed envelopes	No
Pfab et al. (39)	2011	Germany	30	Randomized control trial	Block-random sampling	Patients
Yin et al. (40)	2010	China	28	Randomized control trial	Random number table	No
Han et al. (41)	2010	China	58	Randomized control trial	Random number table	No
Nayak et al. (42)	2008	UK	12	Non-controlled pilot study	-	-
Wu et al. (43)	2007	China	60	Randomized control trial	Random number table	No
Li et al. (44)	2006	China	60	Randomized control trial	Random number table	No

N, Number of patients.

Table 2 provides detailed evidence regarding the particulars of acupuncture therapy in addition to patients' characteristics. Included are details of patients who underwent surgery processes [6 articles: Zhang et al. (18), Liu et al. (19), Bashar et al. (28), Wang et al. (34), Wu et al. (43), Ben-Arie et al. (16)]. EA treatment was implemented as a therapeutic intervention in 12 articles [Xing et al. (15), Xing et al. (17), Liu et al. (19), Zheng et al. (22), Meng et al. (26), Meng et al. (27), Yang et al. (33), Wang et al. (34), Yu et al. (36), Zheng et al. (38), Han et al. (41), Wu et al. (43)], while manual acupuncture was conducted in 7 articles [Ben-Arie et al. (16), Li et al. (23), Feeney et al. (30), Matsumoto-Miyazaki et al. (31), Wang et al. (32), Xiao et al. (35), Li et al. (44)]. Additionally, an acupoint injection, known as a novel technique for acupoint stimulation, was applied in one study [Yin et al. (40)], one study [Zhang et al. (18)] implemented Acupoint Catgut Embedding, and another study [Wang et al. (14)] used Pyonex/epidermal tacks acupuncture. Five studies combined Chinese herbal medicine with acupuncture/EA [Matsumoto-Miyazaki et al. (31), Wang et al. (32), Wang et al. (34), Yin et al. (40), Han et al. (41)].

**Table 2:** Describes the relevant acupuncture-related intervention details of each included study.

**Author (Year)**	**Patient condition**	**Acupuncture type**	**Acupoints**	**Treatment sessions**	**Stimulation duration**	**Control Intervention**	**Follow-up time**
Wang et al. (14)	Undergoing mechanical ventilation	Pyonex/Epidermal tacks acupuncture (Pyonex stickers)	PC6, LI 4 ST36	Every 4 h and repeated every 2–3 days	7 days	ICU care	7 days
Xing et al. (15)	Acute gastrointestinal injury with severe traumatic brain injury	EA	ST36, ST25, ST37, ST39, and CV12	Twice a day for 7 days	30 min	ICU care	7 days, 28-day mortality
Ben-Arie et al. (16)	Post-operative oral cancer and hypopharyngeal cancer	MA	Control group: LI15, SJ 14, LU3, GB35, and BL 59. Interventional group: ST36, ST37, ST39, PC6, and LI4	One time per day for 3 days	20 min	MA (control acupoints)	7 days
Xing et al. (17)	Acute gastrointestinal injury with severe traumatic brain injury	EA	ST36, ST25, ST37, ST39, and CV12	Twice a day (in the morning and in the evening) for 3 days	30 min	ICU care	7 days, 28-day mortality
Zhang et al. (18)	Diabetic patients undergoing open cardiac surgery	Acupoint Catgut Embedding	REN9, ST36, ST28, KID14, and REN6	Chromic catgut strands inserted on the evening before surgery	Long-term (until the strands dissolve in the body)	Sham acupoint catgut embedding (cut but no embedding)	7 days, hospital stay
Liu et al. (19)	Patients after traumatic brain injury surgery	EA	PC 6 and GV 26	Once a day for 14 days	30 min of EA and 60 min needles without EA	ICU care	3 months
Liu et al. (20)	Sepsis of GI dysfunction	TEAS	ST 25, ST 37, ST 41, SP 8, ST 36, CV 12, and SP 15	Twice daily for 5 days	30 min	ICU care	5 days, ICU stay
Wang et al. (21)	ICU-acquired weakness by septic shock	TEAS	GB 30, ST 32, ST 36, GB 39 and LR 3	Twice daily, continued for 7 days	30 min	ICU care	28 days
Zheng et al. (22)	Sepsis-associated encephalopathy	EA	DU20 and DU26	Every 12 h for 1 week	30 min	ICU care	1 week
Li et al. (23)	Sepsis with GI dysfunction	MA	Huatuo Jiaji (EX-B2)	Once a day for 10 days	30 min	ICU care	Not mentioned
Liu et al. (24)	Mechanical ventilation with diaphragmatic dysfunction associated	TEAS	LR3, SP21, BL20, BL23, GB25, SP21, BL17, BL23, REN17, LR14, REN16, REN15, and BL17	Three times a day for 7 days	30 min	ICU care	Hospital stay
AminiSaman et al. (25)	Pneumonia under mechanical ventilation	TEAS	LI4 and ST36	Four times in 24 h	30 min	Placebo transcutaneous electrical acupoint stimulation	28 h
Meng et al. (26)	Sepsis	EA	ST36 and ST37	Twice a day for 5 days	20 min	ICU care	3 days
Meng et al. (27)	Sepsis	EA	ST36 and ST37	Twice a day for 5 days	20 min	ICU care	7 days (the first, the third, and the seventh days)
Bashar et al. (28)	Mechanically ventilated surgical ICU patients with delayed gastric emptying	TENS	PC6, TW5	Twice a day for 6 days	20 min	ICU care	6 days
Bataille et al. (29)	Mix ICU patients	TEA	PC6	One intervention	30 min	No control group	24 h
Feeney et al. (30)	Pain and/or nausea	MA, Ear acupuncture	LI4, LR3, PC6, ST36, and ear acupuncture (Shenmen, Sympathetic, Stomach, and Thalamus)	Once a day for 3 days	20 min	ICU care	Not mentioned
Matsumoto-Miyazaki et al. (31)	Cardiovascular disease	MA and the Chinese herbal formula (mainly Jia-Wei-Gui-Pi-Tang)	GV20, EX-HN3, HT7 or PC6, LI4, LR3, and KI3 or KI7. ST36, ST40, SP9, and LI11 in some patients	Once a day	10 min	ICU care	ICU duration period
Wang et al. (32)	Acute gastrointestinal injury with severe sepsis	MA with TCM herbal therapy	ST36, ST25, ST37, and ST39	Once a day for 7 days	30 min	ICU care	6 days
Yang et al. (33)	Sepsis patients	EA	ST36, REN4	Not mentioned	Not mentioned	ICU care	28-day mortality
Wang et al. (34)	Post-major gastrointestinal surgery	EA with modified *Huang Lian Jie Du* Decoction (TCM)	ST36, ST 37, and ST39	Once a day for 7 days	30 min	ICU care	28-day mortality
Xiao et al. (35)	Sepsis with GI dysfunction	MA	ST36, GB34, PC6, and REN 4	Once a day for 6 days	30 min	ICU care (n=30) Thymosin α1 (*n* = 30)	28-day mortality
Yu et al. (36)	Acute GI injury	EA	ST 36, ST 37, LI 4, LI 11	Once a day for 7 days	30 min	ICU care	7 days
Xiao et al. (37)	Acute heart failure	MA	PC6, HT7, REN17, and other points by patients TCM symptom for yin vacuity: SP6 or KID3 for yang vacuity: REN 4 or DU 14; for qi vacuity: REN 6 or ST36 for Phlegm obstruction ST40; for blood stasis BL17 or SP10	Once a day for 5 days	20 min	ICU care	5 days, 28-day mortality
Zheng et al. (38)	Critically ill with mechanical ventilation	EA	GV24 and EX-HN3	Electrical stimulation with 30-min resting intervals for 6 h	30 min	ICU care	Not mentioned
Pfab et al. (39)	Delayed gastric emptying	TEAS	PC6	One 30-min period and eight 5-min periods per day	30–40 min	Conventional promotility drugs	Not mentioned
Yin et al. (40)	Under mechanical ventilation	Acupoint injection (*Yi Tong Shu*)	ST36	11 h or 23 h after administration of sedative drug	Drug retention without the needle	ICU care	Not mentioned
Han et al. (41)	Acute exacerbation of chronic obstructive pulmonary disease and undergoing non-invasive ventilation	EA with *Xuan Da Cheng Qi* Decoction (TCM)	ST36, ST37, ST40, and LI11	EA—Twice daily	30 min	ICU care	Not mentioned
Nayak et al. (42)	Critically ill patients requiring sedation for mechanical ventilation	TEA	LI4, ST36, HT7, and LR3	20 min every hour for 12 h	20 min every hour for 12 h	No control group	12 h
Wu et al. (43)	Under mechanical ventilation after valve replacement	MA and EA	GV24 and EX-HN3	Every 3 h post-operation till the following morning	30 min	ICU care (injection of propofol)	Not mentioned
Li et al. (44)	Respiratory failure with mechanical ventilation	MA	LI4 and LR3	Every 12 h until MV is discontinued	1 h	ICU care	Not mentioned

In addition to the characteristics pertaining to the patients. EA, Electroacupuncture; TCM, Traditional Chinese Medicine; ICU, Intensive Care Unit; TEA, transcutaneous electroacupuncture; GI, gastrointestinal; MA, Manual acupuncture; TEAS, Transcutaneous electrical acupoint stimulation. Acupoints: LI15 - Jianyu, SJ 14 - Jianliao, LU3 - Tianfu, GB35 - Yangjiao, BL59 - Fuyang, ST25 - Tianshu, ST 32 - Futu, ST36 - Zusanli, ST37 - Shangjuxu, ST39 - Xiajuxu, ST40 - Fenglong, PC6 - Neiguan, LI4 - Hegu, LI11 - Quchi, LR3 - Taichong, LU1 - Zhongfu, LU9 - Taiyuan, LU7 - Lieque, PC4 - Ximen, GV20 - Baihui, GV26 - Shuigou, EX-HN3 - Yin-Tang, HT7 - Shenmen, KI3 - Taixi, KI7 - Fuliu, SP6 - Sanyinjiao, SP9 - Yinlingquan, SP10 - Xuehai, SP21 - Dabao, BL20 - Pishu, BL23 - Shenshu, GB25 - Jingmen, GB 30 - Huantiao, GB34 - Yanglingquan, GB 39- Xuanzhong, SP21 - Dabao, BL17 - Geshu, BL23 - Shenshu, REN4 - Guanyuan, REN 6 - Qihai,REN15 - Jiuwei, REN16 - Zhongting, REN17 - Tanzhong, LR3 - Taichong, LR14 - Qimen, LU10 - Yuji, CV12 - Zhongwan, GV 14 - Dazhui, GV24 - Shenting.

The most commonly used acupoint reported was the Zusanli (ST36) acupoint (21 articles). Meanwhile, Neiguan (PC6) (10 articles) and Hegu (LI4) (7 articles) followed as the next commonly applied acupoints. The frequency of acupuncture treatment conducted once daily was completed in 10 studies [Ben-Arie et al. (16), Liu et al. (19), Li et al. (23), Feeney et al. (30), Matsumoto-Miyazaki et al. (31), Wang et al. (32), Wang et al. (34), Xiao et al. (35), Yu et al. (36), Xiao et al. (37)]. Acupuncture treatment conducted two times a day was reported in 10 studies [Xing et al. (15), Xing et al. (17), Liu et al. (20), Wang et al. (21), Zheng et al. (22), Meng et al. (26), Meng et al. (27), Bashar et al. (28), Han et al. (41), Li et al. (44)]. The remaining frequencies of treatment range in duration and also include continuous stimulation (such as Pyonex/epidermal tack needles or acupoint catgut embedding). In addition, treatment duration in all 31 publications ranged from 1 intervention up to as long as it took until MV was discontinued. The most commonly reported treatment duration time was 3 days, which was reported in two articles [Feeney et al. (30), Ben-Arie et al. (16)]; 5 days, which was reported in four articles [Liu et al. (20), Meng et al. (26), Meng et al. (27), Xiao et al. (37)]; and 7 days, which was reported in seven studies [Wang et al. (14), Wang et al. (21), Zheng et al. (22), Liu et al. (24), Wang et al. (32), Wang et al. (34), Yu et al. (36)]. The most common needle retention time recorded in each session was at least 30 min (see Table 2).

### Risk of bias

**Figure 2:**
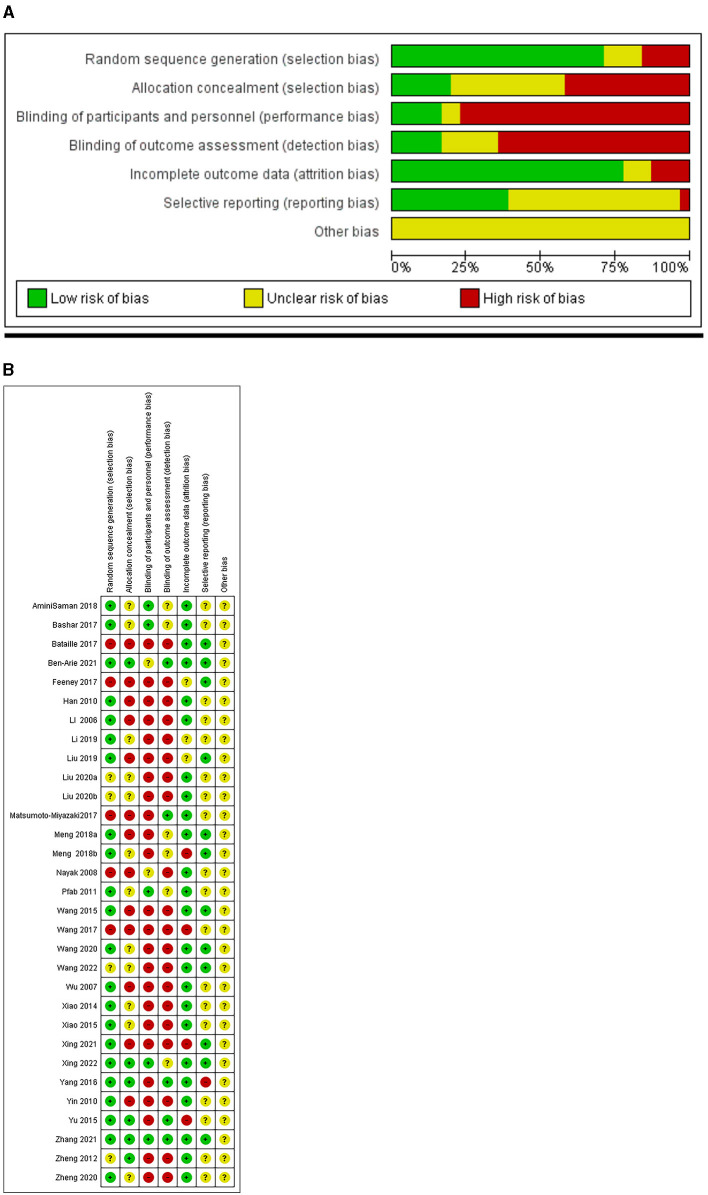
**(A)** Weighted bar plots of the distribution of risk-of-bias judgements within each bias domain. **(B)** “Traffic light” plots depicting the risk of bias in the included studies in accordance with the Cochrane collaboration's tool for assessing risk of bias in randomized trials.

Regarding the risk of bias, in randomization, four studies were rated as unclear (14, 19, 20, 38), and five studies were rated as high risk of bias (29–32, 42). For group allocation, six studies reported the group allocation methods with sufficient details and were ranked as having a low risk of bias (15, 16, 18, 33, 36, 38), whereas the remaining studies did not report the allocation processes in a proper way. Only five studies reported adequate and correct patient and personal blinding (15, 18, 25, 28, 39). The outcomes assessment blinding was well reported in five studies (16, 18, 31, 33, 36). For incomplete outcome data, three studies were ranked as unclear (23, 24, 30), and four studies were ranked as high risk of bias (17, 27, 32, 36). Selective reporting was rated as an unclear risk of bias in 18 studies for not reporting AEs, among other reasons. Furthermore, one study was ranked as having a high risk of bias (33). All studies were ranked as unclear risk of bias for other bias due to the following reasons: changes to study protocol, small sample size, a significant difference in baseline characteristics between groups, missing data, same IRB number as another study, unclear funder information, unequal sample size, and unclear follow-up time (Figure 2).

### Adverse events

**Figure 3:**
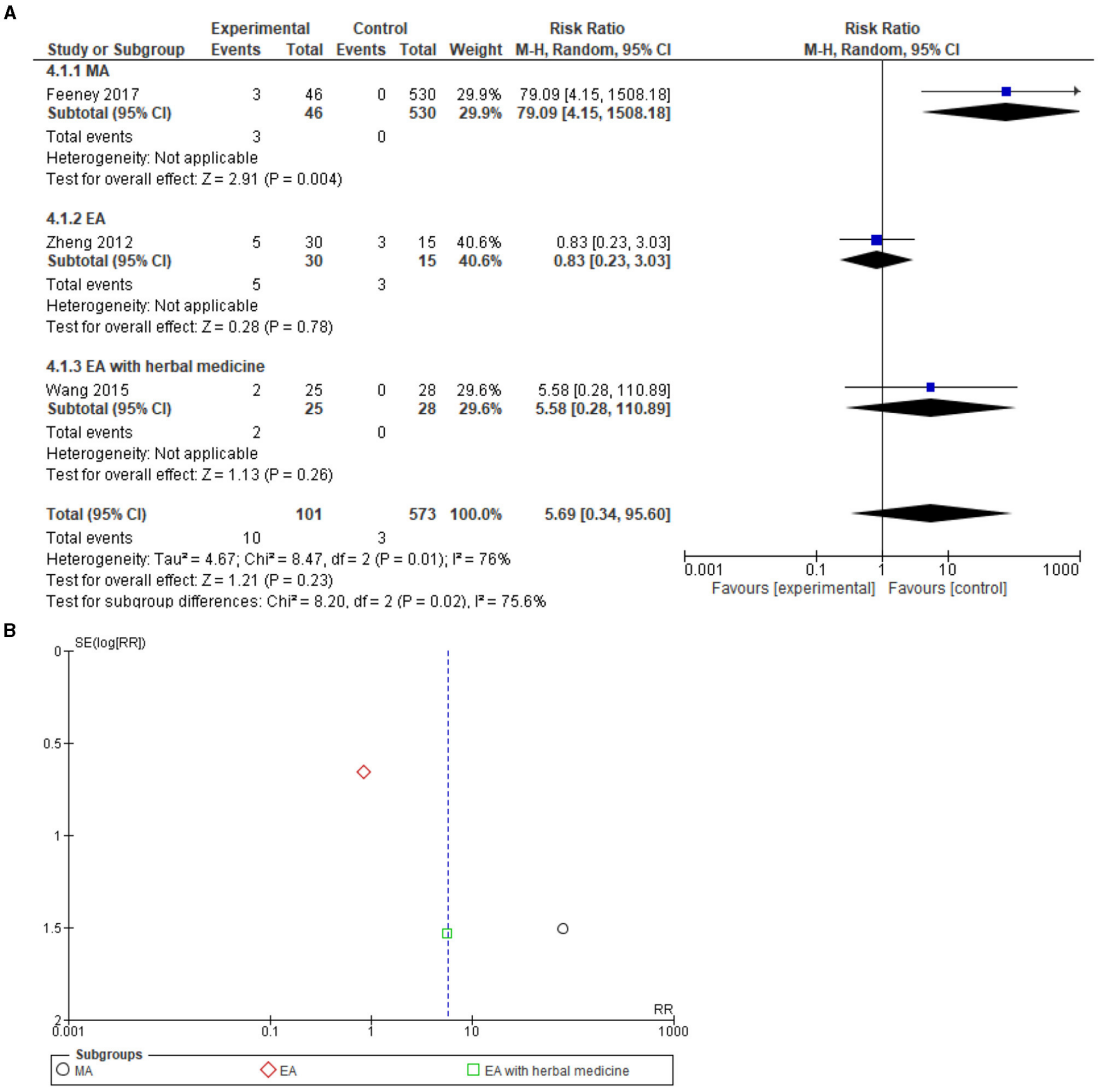
**(A)** Forest plot of 3 studies on minor adverse events. No serious AEs correlated to acupuncture. **(B)** Funnel plot for the analysis. M-H, Mantel-Haenszel test; CI, confidence interval; EA, Electroacupuncture; MA, Manual acupuncture; TEAS, Transcutaneous electrical acupoint stimulation.

Table 3 reveals a comparison between 2,947 patients from 31 studies (1,251 of the interventional group and 1,696 of the control group) and evaluates the reporting of the AEs. No serious AEs following acupuncture treatment were reported in any of the included studies. For minor AEs, only 3 out of the 22 included studies reported minor AEs following acupuncture [Feeney et al. (30), Wang et al. (34), and Zheng et al. (38)]. There were a total of 10 patients from the acupuncture groups in the included studies who reported minor AEs resulting from acupuncture interventions. However, a record of AEs in control groups for three patients in the included studies was also revealed (Figure 3). Feeney et al. (30) showed that 3 out of 46 patients in the acupuncture group described minimal AEs of needling insertion pain, agitation on the first day of treatment, and nausea on the second day of treatment. Wang et al. (34) presented 2 of 25 patients in the EA groups that reported diarrhea. In the study conducted by Zheng et al. 5 patients out of 50 allocated to the EA treatment group had AEs, 2 cases of hypotension, and 3 cases reported abdominal distention after acupuncture treatment. Regarding the methodology of Wang et al. (32), which provided details of withdrawal from the experiment under the condition of the occurrence of an AE in the study's method section. Eventually, a total of 29 patients dropped out of the acupuncture group in the study without citing any reason, although the possibility of AEs potentially being the reason for the high dropout rates cannot be ruled out.

**Table 3:** The table describes a comparison of adverse events, ICU stay, and mortality events in 2947 patients across the 31 included studies.

**Author (year)**	**Control group (n)**	**Acupuncture group (n)**	**AEs (control)**	**AEs (acupuncture)**	**ICU stay (control)**	**ICU stay (acupuncture)**	**Mortality events (control)**	**Mortality events (acupuncture)**	**Adverse and mortality event details**
Wang et al. (14)	44	44	Adverse reaction: 35	Adverse reaction: 9	22.66 ± 12.91	14.98 ± 7.04	21	10	Hypotension: ACU = 2, CON = 10. Respiratory depression: ACU = 2, CON = 11. Vomiting: ACU = 2, CON = 6 Delirium: ACU = 0, CON = 4 Bradycardia: ACU = 3, CON = 4 Mortality follow-up: not specific.
Xing et al. (15)	250	250	0	0	16.46 ± 6.03	15.44 ± 5.41	83	57	Mortality follow-up: 28 days.
Ben-Arie et al. (16)	0	26	0	0	-	-	0	0	-
Xing et al. (17)	50	50	0	0	19.26 ± 5.42	16.76 ± 4.68	16	11	Mortality follow-up: 28-days. The patients in the acupuncture group had no significant adverse reactions to EA treatment.
Zhang et al. (18)	46	46	0 adverse reaction: 21	0 adverse reaction: 11	24.3 ± 5.7	22.1 ± 4.7	0	0	The study reports no adverse events. However, in the text, they described: Infections: ACU = 2, CON = 5, Acute kidney injury: ACU = 5, CON = 6, PONV: ACU = 4, CON = 10.
Liu et al. (19)	50	50	Not reported	Not reported	-	-	6	5	Mortality follow-up time not specific.
Liu et al. (20)	25	24	Not reported	Not reported	4.92 ± 0.70	3.54 ± 0.66	-	-	-
Wang et al. (21)	28	27	0	0	14.7 ± 4.4	13.1 ± 2.8	5	4	Mortality follow-up: 28 days.
Zheng et al. (22)	35	35	Not reported	Not reported	-	-	0	0	-
Li et al. (23)	20	20	Not reported	Not reported	-	-	-	-	-
Liu et al. (24)	26	23	Adverse reaction: Reintubation: 8	Adverse reaction: Reintubation: 1	6.92 ± 1.84	5.78 ± 1.45	3	2	Mortality follow-up time: not specific. Adverse reactions: re-intubated: ACU = 1 (due to carbon dioxide retention) CON = 8 (due to cough and pulmonary infection N = 3 type II respiratory failure N = 3, aggravated intubation and drainage N = 4).
AminiSaman et al. (25)	25	25	Not report	Not report	-	-	-	-	-
Meng et al. (26)	41	41	0	0	14.02 ± 2.66	13.66 ± 2.81	16	14	Mortality follow-up: 28 days.
Meng et al. (27)	36	35	0	0	13.9 ± 2.7	13.6 ± 2.8	15	13	Mortality follow-up: 28 days.
Bashar et al. (28)	30	30	Not report	Not report	Not report	Not report	-	-	-
Bataille et al. (29)	No control	10	No control	0 adverse reaction: 5 patients	-	-	-	-	Adverse reaction: Nausea (6 hours after TEA): TEA = 2 Required antiemetic rescue: TEA = 3 Required surgical procedure: TEA = 2. The study report: there were no complications or side effects related to TEA.
Feeney et al. (30)	530	46	No report	3	4.62 ± 5.96	4.42 ± 4.26	-	-	Slight pain with needle insertion: ACU = 1 Agitation during the treatment: ACU = 1 (persisting for 10 minutes) Worsening nausea and requested to be withdrawn from the study: ACU = 1. 16 over 909 needles fell out during treatment. Vital signs post interventions were not significant between the groups.
Matsumoto-Miyazaki et al. (31)	29	30	0	0	Original report: 4 (3, 8) 95% CI Converted to 4 ± 10.5 by RevMan calculator	Original report: 4 (3, 6) 95% CI Converted to 4 ± 5.4 by RevMan calculator	5	3	ICU mortality: ICU mortality: ACU = 2, CON = 3 Hospital mortality: ACU = 3, CON = 5. The mortality circumstance has no direct association with acupuncture treatment
Wang et al. (32)	150	146	Did not report	Did not report (29 patients drop out)	18.5 ± 10.6	14.2 ± 7.9	65	46	Mortality follow-up: 28 days: Study methods noted: “If an adverse event was suspected of resulting from the TCM intervention, the investigator had to fill the form for adverse events and drop the patient out of the study.” The intervention group had 29 patients lost to follow-up without citing any reasons.
Yang et al. (33)	29	29	No report	No report	-	-	9	5	Mortality follow-up: 28 days
Wang et al. (34)	28	25	0	2	6 ± 3	5 ± 3	1	1	Mortality follow-up: 28 days. Diarrhea: ACU *=* 2. In addition, EA patients also received Chinese herbal medicine. The mortality circumstance has no direct association with acupuncture treatment.
Xiao et al. (35)	ICU care: 30 thymosin α1: 30	30	No report	No report	ICU care: 7.9 ± 1.6; thymosin: 6.2 ± 1.7	6.0 ± 1.5	ICU care: 9, thymosin: 6	5	28 days mortality
Yu et al. (36)	10	10	No report	No report	-	-	-	-	The study results did not mention adverse events. The study methods mention: “Patients reporting severe adverse effects were also excluded” it's unclear if there were severe adverse events or not.
Xiao et al. (37)	30	30	No report	No report	7.8 ± 1.9	5.6 ± 1.8	9	5	Mortality follow-up: 28 days
Zheng et al. (38)	15	30	3	5	-	-	0	0	Hypotension: ACU = 2, CON 2 Abdominal distention: ACU = 3 Vomiting: CON = 1.
Pfab et al. (39)	15	15	No report	No report	-	-	-	-	-
Yin et al. (40)	14	14	No report	No report	-	-	-	-	-
Han et al. (41)	30	28	No report	No report	-	-	-	-	-
Nayak et al. (42)	0	12	No report	No report	-	-	-	-	-
Wu et al. (43)	20	40 (2 treatment groups)	No report	No report	-	-	-	-	-
Li et al. (44)	30	30	No report	No report	-	-	-	-	-
**Total**	**1,696**	**1,251**	**3**	**10**			**263**	**181**	

ICU, Intensive Care Unit; TEA, transcutaneous electroacupuncture; ACU, Acupuncture group; CON, Control group; PONV, Postoperative nausea and vomiting; EA, Electroacupuncture.

A total of three studies, Feeney et al. (30), Wang et al. (34), and Zheng et al. (38) reporting minor AEs, were included in a meta-analysis that revealed a risk ratio of 5.69 [0.34, 96.60], with high heterogeneity *I*^2^ = 76%, and test for overall effect: Z = 1.21 (*P* = 0.23). No significant differences were found between the acupuncture and the control groups (Figure 3). Funnel plot analysis showed no asymmetry and suggested no publication bias. In addition, Egger's test result was recorded as *T* = 1.84, *P* = 0.29.

### Adverse reactions

A total of 26 adverse reactions were reported in the acupuncture groups of four studies [Wang et al. (14), Zhang et al. (18), Liu et al. (24), Bataille et al. (29)], and 64 adverse reactions were reported in the control groups of three studies [Wang et al. (14), Zhang et al. (18), Liu et al. (24)]. The adverse reactions include hypotension, respiratory depression, vomiting, delirium, bradycardia, infections, acute kidney injury, nausea, vomiting, and the need for reintubation (Table 3).

**Figure 4:**
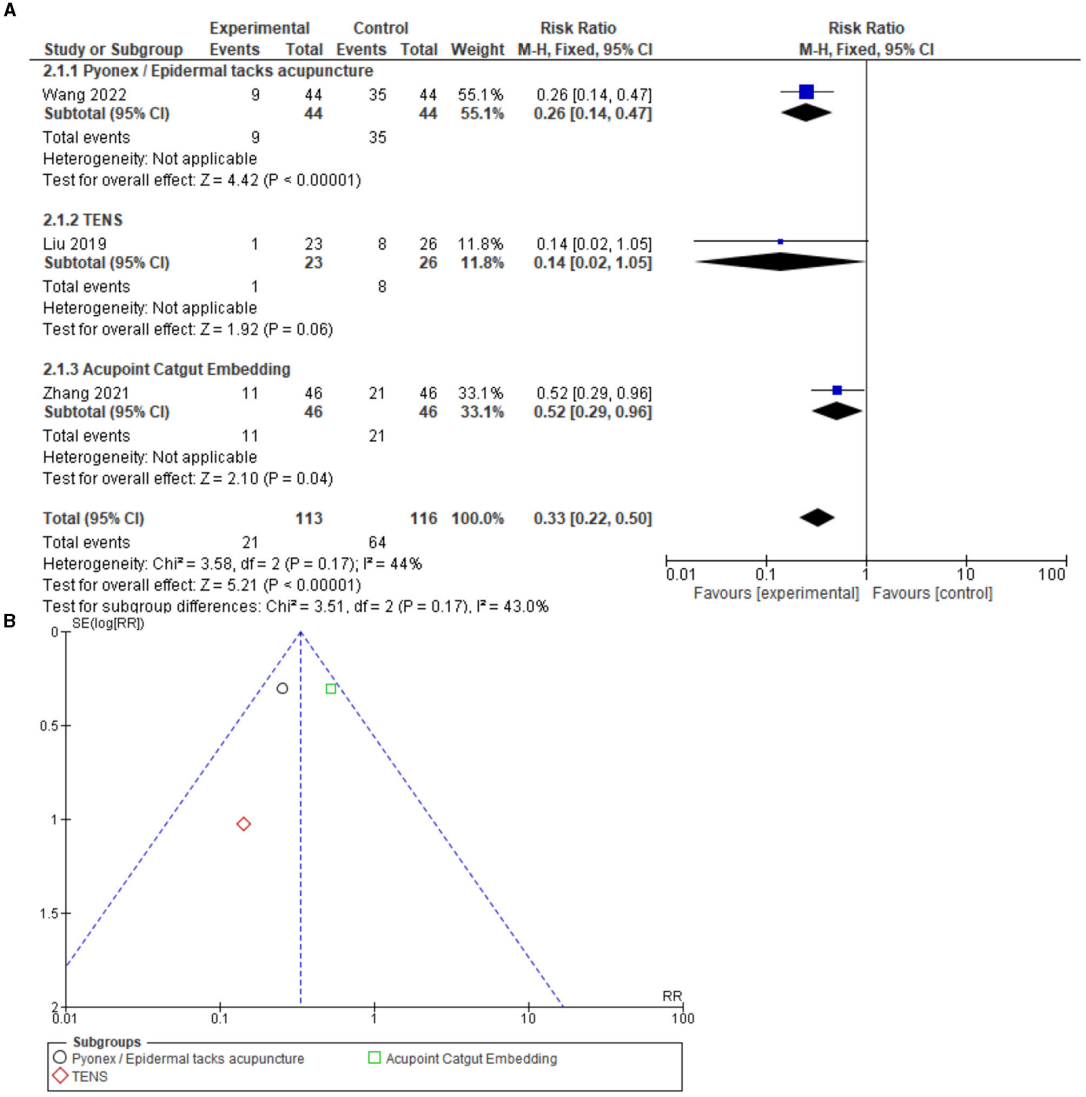
**(A)** Forest of 3 studies on adverse reactions. Significantly fewer adverse reactions correlated to acupuncture compared to the control. **(B)** Funnel plot for the analysis. M-H, Mantel-Haenszel test; CI, confidence interval; EA, Electroacupuncture; MA, Manual acupuncture; TEAS, Transcutaneous electrical acupoint stimulation.

In a meta-analysis including adverse reactions comparing acupuncture to the control (ICU care), a total of three studies [Zhang et al. (18), Wang et al. (14), Liu et al. (24)] were included, and the results indicate a risk ratio of 0.33 [0.22, 0.50] 95% CI, test for overall effect: *Z* = 5.21 (*P* = 0.00001), and heterogeneity *I*^2^ = 44%, which reveals a significantly reduced risk for adverse reactions following acupuncture when compared to the control (Figure 4). Funnel plot analysis showed no asymmetry and suggested no publication bias. In addition, Egger's test result was recorded as T = 0.55, *P* = 0.68.

In a meta-analysis including AEs or adverse reactions comparing acupuncture to the control (ICU care), a total of six studies [Feeney et al. (30), Wang et al. (34), Zheng et al. (38), Zhang et al. (18), Wang et al. (14), Liu et al. (24)] were included, and the results indicated a risk ratio of 0.74 [0.27, 2.04] 95% CI, with a test for overall effect: Z = 0.58 (*P* = 0.56) and heterogeneity *I*^2^ = 76%, indicating no significant difference between the groups. The funnel plot visual analysis indicated no asymmetry. Therefore, publication bias is not suspected. In addition, Egger's test was recorded as T = 1.65, *P* = 0.17 (Supplementary Figure 1).

This systematic review and meta-analysis included all the possible publications that used acupuncture in the ICU, regardless of whether or not the study reported any AEs. Hereby, the overall safety of acupuncture in the ICU setting could be examined. A total of 11 studies were included to assess the overall safety of acupuncture, including studies that reported zero AEs. The meta-analysis displayed a risk difference of −0.07 [−0.15, 0.02] 95% CI, test for overall effect: Z = 1.55 (*P* = 0.12), and heterogeneity *I*^2^ = 96%, which subsequently showed no significant differences between control and treatment groups in terms of overall safety. The funnel plot visual analysis did not indicate major asymmetry, which subsequently suggests that publication bias is not suspected. In addition, Egger's test was reported as T = 1.27, *P* = 0.23 (Supplementary Figure 2).

### ICU stay

**Figure 5:**
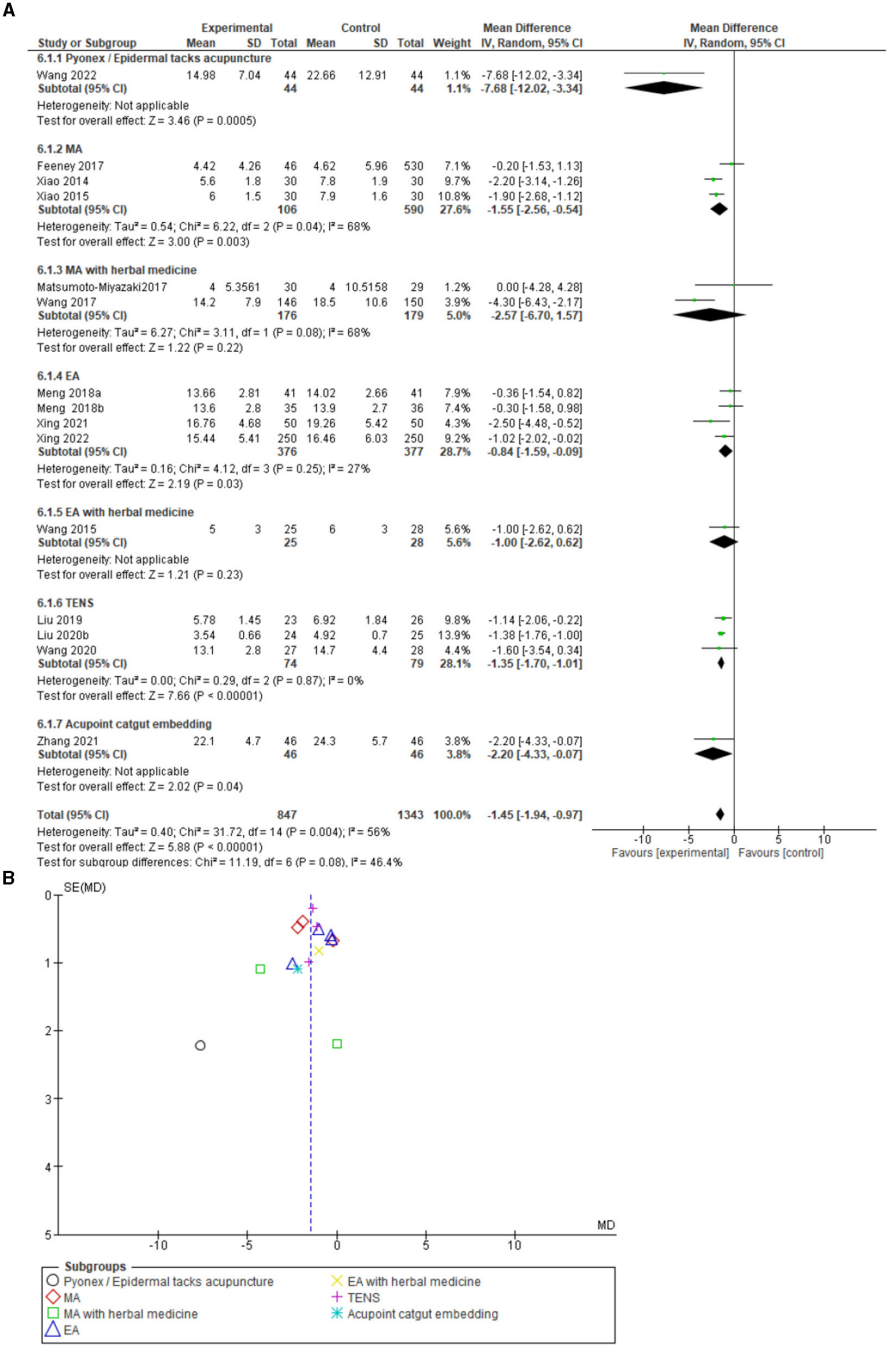
**(A)** Forest plot including 15 studies on ICU stay. **(B)** Funnel plot for the analysis. SD, standard deviation; CI, confidence interval; IV, inverse variance; EA, Electroacupuncture; MA, Manual acupuncture; TEAS, Transcutaneous electrical acupoint stimulation.

The amount of time that patients spent in the ICU was also investigated. A meta-analysis showed a significant reduction in ICU days in the acupuncture arm compared to the control arm (ICU care), which included 15 studies. The analysis discovered a mean difference of −1.45 [−1.94, −0.97] 95% CI, test for overall effect: Z = 5.88 (*P* = 0.00001), and heterogeneity *I*^2^ = 56% (Figure 5). The funnel plot analysis showed no major asymmetry. Therefore, publication bias is not suspected. In addition, Egger's test revealed T = 0.79, *P* = 0.44.

### Mortality

A total of 14 studies reported mortality cases (different follow-up periods) in both the control and experimental groups [Wang et al. (14), Xing et al. (15), Liu et al. (19), Wang et al. (21), Yang et al. (33), Xiao et al. (35), Xiao et al. (37), Meng et al. (26), Meng et al. (27), Matsumoto-Miyazaki et al. (31), Liu et al. (24), Wang et al. (34), Wang et al. (32), and Xing et al. (17)]. Mortality data, which were reported in this review, showed 181 patients in the acupuncture arm vs. 263 in the control arm. However, no record regarding serious AEs leading to death was reported, and all of the associated mortality circumstances did not have any direct relation to the administration of acupuncture therapy.

A combined mortality meta-analysis (including ICU, hospital, and 28-day mortality), which included 14 studies, revealed a significant reduction in mortality in the acupuncture arm compared to the control arm (ICU care) with an odds ratio of 0.60 [−0.47, 0.75] 95% CI, test for overall effect: Z = 4.48 (*P* = 0.00001), and no heterogeneity *I*^2^ = 0%. The funnel plot analysis showed no asymmetry and suggested no publication bias. In addition, Egger's test was recorded as T = 0.31, *P* = 0.76 (Supplementary Figure 3).

**Figure 6:**
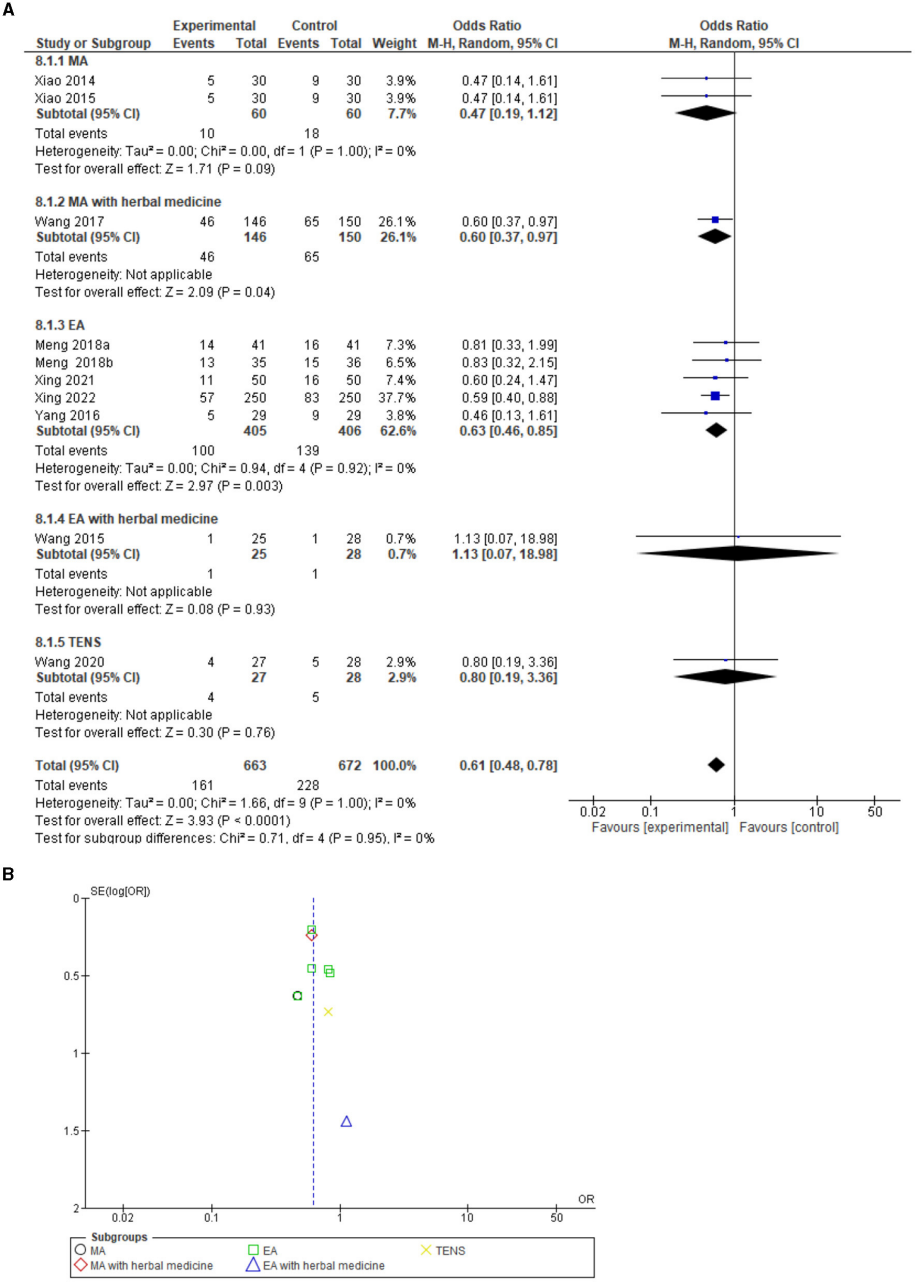
**(A)** Forest plot including 10 studies on 28-day mortality. **(B)** Funnel plot for the analysis. M-H, Mantel-Haenszel test; CI, confidence interval; EA, Electroacupuncture; MA, Manual acupuncture; TEAS, Transcutaneous electrical acupoint stimulation.

For 28-day mortality, which included 10 studies, a significant 28-day mortality reduction following acupuncture was identified, with an odds ratio of 0.61 [0.48, 0.78] 95% CI, test for overall effect: Z = 3.93 (*P* = 0.0001), and no heterogeneity *I*^2^ = 0% (Figure 6). The funnel plot analysis showed no asymmetry and suggested no publication bias. In addition, Egger's test was recorded as T = 0.55, *P* = 0.595.

With regard to the GRADE rating for the certainty of evidence in each meta-analysis, both minor AEs and the ICU stay meta-analyses were ranked at a very low level of certainty due to a high risk of bias, heterogeneity, and a small sample size. The meta-analysis on adverse reactions was ranked as having a low level of certainty due to the high risk of bias and small sample size. The meta-analysis on 28-day mortality was ranked as having a moderate level of certainty in the results due to some risk of bias (Supplementary Table 1).

### Sensitivity analysis

A sensitivity analysis regarding the minor AEs (Figure 3) and meta-analysis resolved the high heterogeneity after excluding the Feeney et al.'s (30) study possibly due to the study's unequal sample size being of importance in this regard (heterogeneity: Tau^2^ = 0.54; chi^2^ = 1.39, df = 1, *P* = 0.24; *I*^2^ = 28%, 95% CI 1.35 [0.25, 7.17], test for overall effect: Z = 0.35, *P* = 0.73). With regard to the meta-analysis of ICU stay in days (Figure 5), the high heterogeneity was resolved after excluding the Wang et al. (14) study and the Wang et al. (32) study (heterogeneity: Tau^2^ = 0.10; chi^2^ = 16.34, df = 12, P = 0.18; I^2^ = 27%, 95% CI −1.30 [−1.65, −0.94], test for overall effect: Z = 7.07, *P* < 0.00001).

### Publication bias

For unpublished work, there are three ongoing studies that were found in the http://clinicaltrials.gov/ search. Specifically, two studies are affiliated with the China Medical University Hospital and our research team. Another study with unknown status is affiliated with the Jiangsu Province Hospital of Traditional Chinese Medicine. In addition, a visual examination of the funnel plot asymmetry derived from the meta-analyses conducted did not indicate substantial publication bias, with the exception of Supplementary Figure 2, where asymmetry is indeed suspected. However, Egger's test was not significant for small study effects across all of the meta-analyses (*P* > 0.05).

## Discussion

This systematic review and meta-analysis examined the overall safety and tolerance of acupuncture in both medical and surgical ICUs. The categories of acupuncture use included EA, manual acupuncture, transcutaneous electrical stimulations, and so on as an additional treatment to the regular routine ICU care. The main findings of this study reveal that no severe AEs, such as major bleeding or pneumothorax, were reported following acupuncture treatment in an ICU setting. However, minor AEs were observed to occur more frequently in the acupuncture group, which forms part of a non-significant trend. In the majority of the included studies, AEs are not incorporated as primary or even secondary outcome measurements. Hence, this investigation, which includes a total of 31 papers, consisted of 7 separate studies that effectively only described minor AEs or adverse reactions following acupuncture intervention in a total of 224 participants. However, when compared to the routine ICU care control arm, acupuncture did not cause more harm. With regard to other measurements, a statistically significant reduction in ICU stay and 28-day mortality for patients in the acupuncture groups compared to the control was observed.

AEs are defined as injurious or detrimental outcomes that occur during or after the implementation of a drug or intervention but are not necessarily caused by it (45). The reported information on these circumstantial occurrences is extremely important to regulators, policymakers, healthcare professionals, and patients alike. Given that serious AEs in acupuncture occur in rare instances, accurate reporting of these events is fundamentally significant to provide greater insights while maintaining patient safety (46).

To the best of our knowledge, this is the first study to analyze the safety of acupuncture-related treatment in critically ill patients in an intensive care setting through a systematic review and meta-analysis, with the associated outcome results being related to the incidence of AEs. Given the affiliated understanding of AEs, when considering the related potential harms accompanying patients receiving acupuncture treatment, the prevalence of a pneumothorax is perhaps the most important when observing patients, both inside and outside the ICU (47). The incidence of pneumothorax was recorded as 2 out of 229,230 patients, with an average of 10 interventions per person in the outpatient department, as determined by a large observational study (8). From a technical point of view, visceral injury caused by acupuncture, including pneumothorax, could be prevented by the caution of needle depth, avoiding trunk points, and the use of different acupoint stimulation methods such as TENS or acupoint pressure, etc.

The most common AEs from acupuncture treatments are minor hemorrhage, pain, and vegetative symptoms, presenting with a total incidence of 9.31% per 100 patients after receiving a series of acupuncture treatments (8, 48). The occurrence of severe AEs is rare, with an incidence estimation of 1.01 per one million treatments (48). Within an ICU setting, the emphasis on the severity of known acupuncture-related AEs allows practitioners to safely navigate the treatment process without further causing injury to the patient. The presentation of evidence for acupuncture treatment directs focus on how to avoid harm in an ICU setting through appropriate clinical application. For example, the acupuncture treatments in the ICU should practically take into consideration the insertion depth of the needle, needling of the extremities instead of the truncal region, avoiding injured areas that may be bandaged, bruised, or burned, and considering the ICU environment with regard to space, machines, needles, tubes, and so on. Also, the vital signs need to be closely monitored during the treatment, with medical staff acting accordingly should the need arise (49). The measurement of physical function and response in ICU patients is imperative to allow for successful treatment. Hence, observing certain limitations allows for improved intervention efficacy as well as enriched recovery outcomes.

Given the medically recognized safety of acupuncture treatment, it is understood that the most commonly affiliated AEs are not abnormal but form an integral part of the treatment process (50). Given the inconsequential nature of the categorized minor AEs recorded, including common indications of nociceptive reactions and marginal needle insertion area bleeding, some of these AEs can be considered to be part of the therapeutic process. Given this logical approach, one can identify how the pain of an injection could not necessarily be considered a harmful side effect but rather a part of the treatment that can be recognized and understood. Also, in this systematic review and meta-analysis, Wang et al. reported two cases of diarrhea; those cases can possibly be associated with the herbal medicine combination used in this study in addition to EA (34). Zheng et al. reported acupuncture AEs in both the acupuncture group and control group as abdominal distension and hypotension episodes. Due to the study design of Zheng et al., the AEs could be related to the sedative agents (midazolam) rather than acupuncture. It is highly possible that AEs are underreported in the included studies as 16 studies did not include an AEs section in their study. This high number of studies is alarming and calls for an improvement in awareness of AEs reporting in future acupuncture studies. It is also likely that very minor AEs such as local pain at the needling site and slight bleeding after needle removal were ignored by the researchers and potentially considered to be unimportant and thus not reported as these signs often follow acupuncture interventions. Furthermore, local pain at the needling site and slight bleeding are not considered AEs in other interventions that include needles, such as intravenous therapy.

The systematic review and meta-analysis found a significant reduction in ICU stay, detailed as a 1.45-day reduction following acupuncture. This finding suggests that acupuncture might not only be safe but also improve the outcome of ICU patients. The reduction in ICU stay is supported by the Asmussen et al. meta-analysis findings that also found a reduction in ICU stay following electroacupuncture for critically ill patients with cardiac anesthesia (51). When observing the incidence of mortality, this study presents a total of 810 patients in the acupuncture groups, of whom 181 succumb to their medical conditions, not as a direct result of the treatment but rather as a result of the deterioration of their medical condition. Moreover, the control groups consisted of a total of 821 patients, of whom 263 succumbed to their condition. The reduction in mortality was significant following acupuncture. Ten studies reported patient mortality data that was either hospital mortality or 28-day mortality. The significant reduction in mortality does allude to the fact that acupuncture treatment might provide survival benefits. However, two of the five studies included a combination of acupuncture and herbal medication use, and therefore the mortality reduction findings should also consider the effect of Chinese herbal medicine. In the literature, the association between acupuncture and severe disease-improved survival in large-scale studies has yet to be investigated. Therefore, future studies should investigate the impact of acupuncture on severe disease mortality.

The limitations of this study include a limited sample size. In some studies, the mortality data were presented as a percentage, which was then converted to a number by calculating the relative percentage in relation to the study sample size. Furthermore, AEs were not clearly reported in some studies, and AEs were not reported as major outcome measurements in the included studies. Then, the level of certainty in the evidence ranged from very low to moderate in the meta-analyses. Meng et al. (26) and Meng et al. (27) have the same IRB approval number, and it is unclear if the reported patients in each study are the same patients assessed under different circumstances or if they are different patients. In this regard, an attempt to contact the authors was unsuccessful.

This study concludes that, from the currently available evidence, no serious AEs associated with acupuncture in critically ill patients were identified. The acupuncture arm had more minor adverse events cases, but not statistically significant. Yet, the acupuncture arm had significantly less adverse reactions compared to the control. This work also found a significant reduction in ICU stays and 28-day mortality following acupuncture. However, the results of this systematic review and meta-analysis should be interpreted with caution as the AEs might be under-reported in acupuncture studies, and this work included a limited number of patients. Standardized AE assessment tools, with clear criteria for differentiating acupuncture-related AEs from common therapeutic reactions, are necessary, and the subsequent identification of patient-related risk factors for AEs, not only in an ICU setting but throughout all of the related acupuncture studies, is warranted.

## Data availability statement

The original contributions presented in the study are included in the article/Supplementary material, further inquiries can be directed to the corresponding authors.

## Author contributions

EB-A, P-YK, and Y-CL designed the study. EB-A, P-YK, and BL drafted the manuscript. EB-A, P-YK, and CI contributed to the data collection and analysis of the data. EB-A and W-CH helped in data analysis. CI, BL, EB-A, P-YK, and Y-CL revised the manuscript. Y-CL and F-PC contributed to supervision. All authors have read and approved the final manuscript.
