# Supplementary material for: Is acupuncture safe in the ICU? A systematic review and meta-analysis
Source: Front Med (Lausanne). 2023 Aug 24;10:1190635. doi: 10.3389/fmed.2023.1190635 (PMC10484589; doi:10.3389/fmed.2023.1190635)
Supplement: Supplementary file 1 [file Data_Sheet_1.docx]

**Supplementary Figure 1: Adverse events or adverse reactions meta-analysis**

**
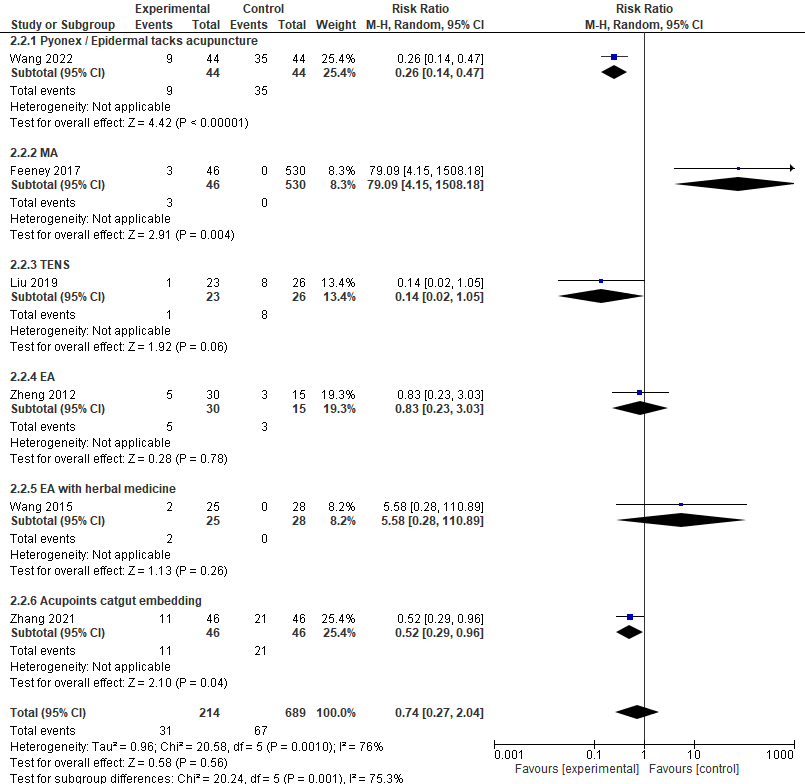
A**

**
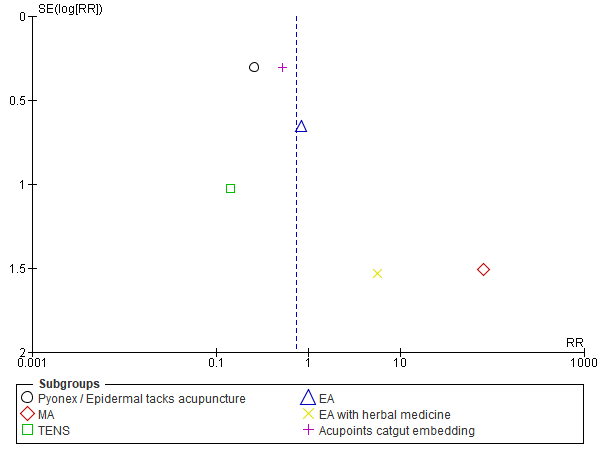
B**

A:Forest plot of 6 studies on adverse events or adverse reactions. No serious AEs correlated to acupuncture. M-H=Mantel-Haenszel test, CI = confidence interval, EA – Electroacupuncture, MA - Manual acupuncture, TEAS - Transcutaneous electrical acupoint stimulation.. * B: Funnel plot for the analysis, Egger’s test (2 tails): T=1.65, P=0.17

Sensitivity analysis was done in order to discover the source of the high heterogeneity, however, the source of the heterogeneity was not detected.

**Supplementary Figure 2:** **Overall safety in the included studies.**

**
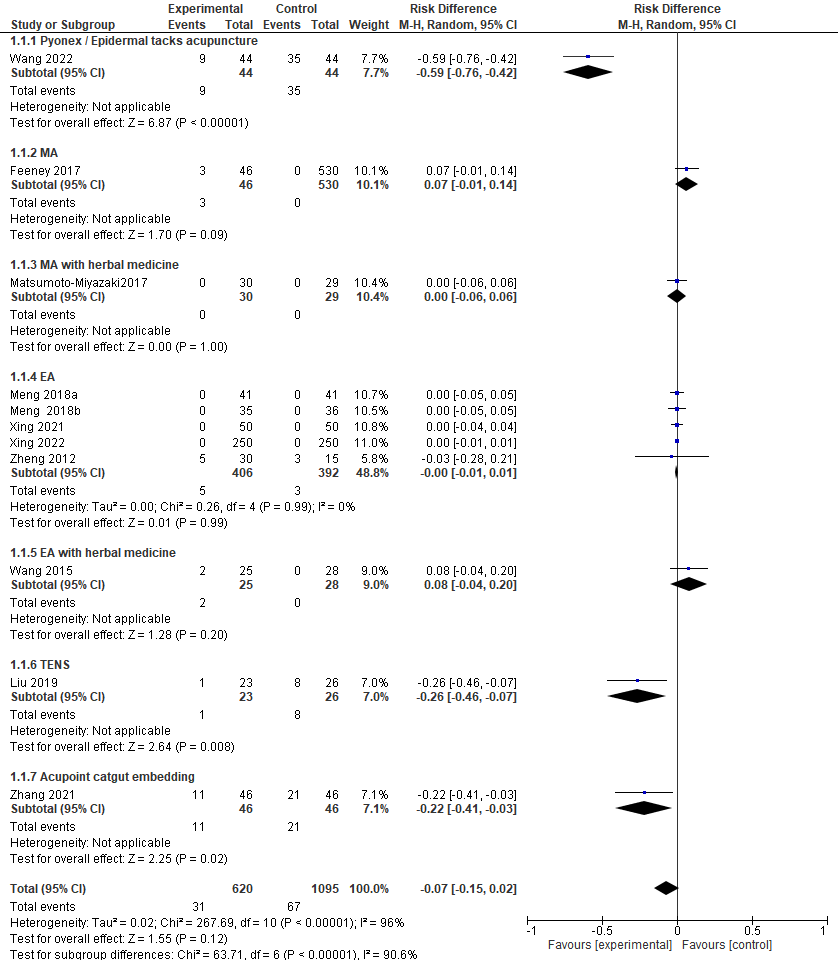
A** **
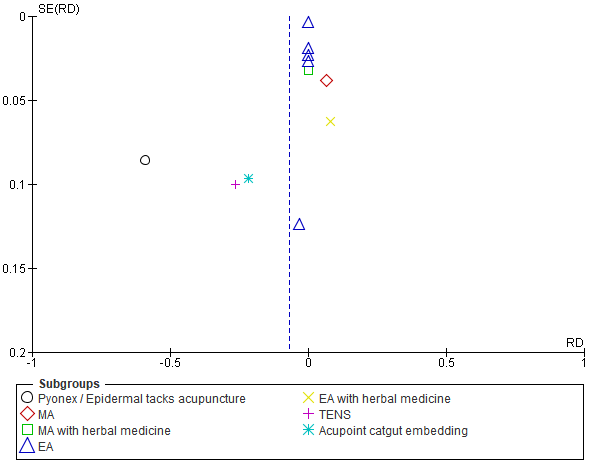
B**

A: Forest plot including 11 studies on the overall safety of the interventions. Adverse events or adverse reactions reported (include 0 cases). No serious AEs correlated to acupuncture. M-H= Mantel-Haenszel test, CI = confidence interval, EA – Electroacupuncture, MA - Manual acupuncture, TEAS - Transcutaneous electrical acupoint stimulation. B: Funnel plot for the analysis, Egger’s test (2 tails): T=1.27, P=0.23.

A sensitivity analysis on Overall safety meta-analysis resolved the high heterogeneity after excluding Liu et al. 2019 study and Wang et al. 2022 (Heterogeneity: Tau² = 0.00; Chi² = 11.98, df = 8, P = 0.15; I² = 33%, 95% CI 0.00 [-0.02, 0.02], Test for overall effect: Z = 0.34, P = 0.74).

**Supplementary Figure 3: Mortality (combined follow-up) in the included studies.**


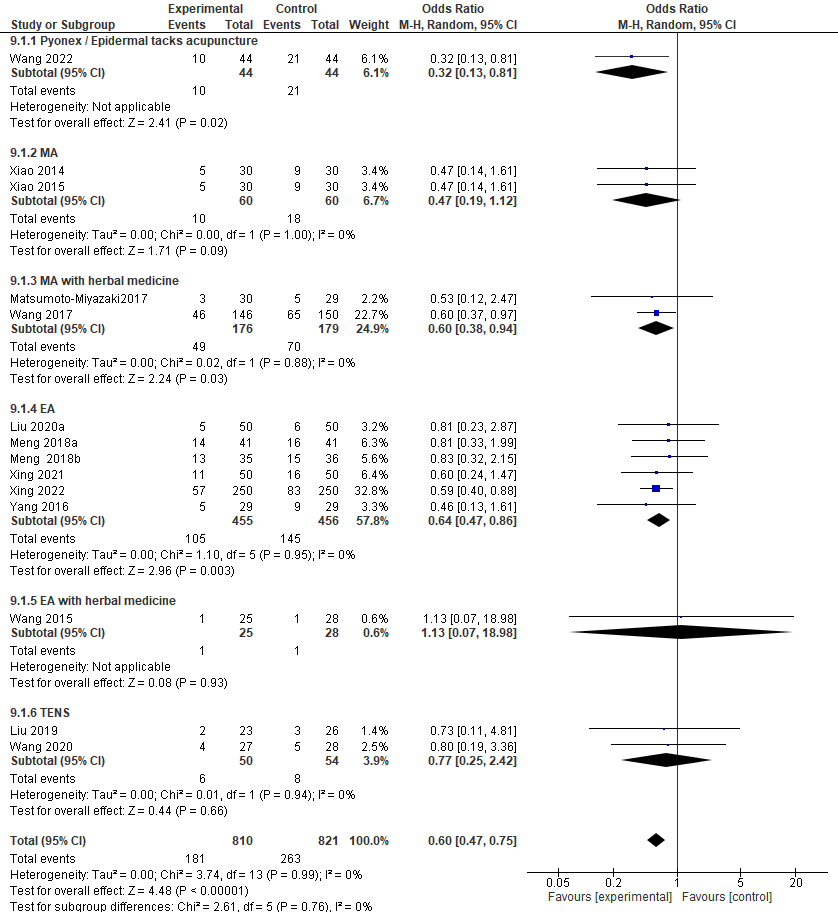
**A** **
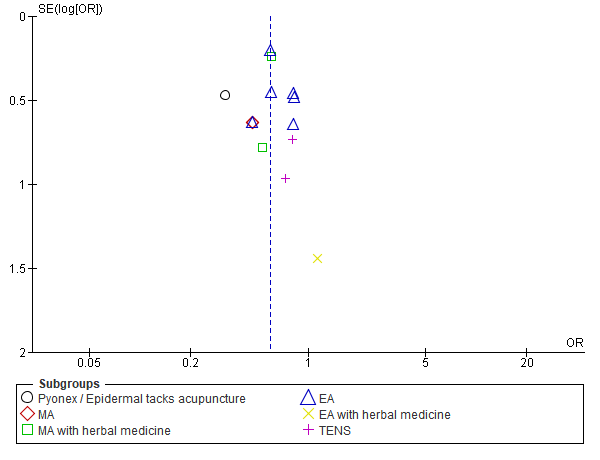
B**

A: Forest plot including 14 studies on mortality follow-ups including ICU, hospital, and 28 days mortality. M-H= Mantel-Haenszel test, CI = confidence interval, EA – Electroacupuncture, MA - Manual acupuncture, TEAS - Transcutaneous electrical acupoint stimulation. B: Funnel plot for the analysis, Egger’s test (2 tails): T=0.31, P=0.76.

**Supplementary Table : GRADE Summary of Findings**

**Patient or population:** ICU patients

**Setting:** ICU

**Intervention:** Acupuncture + ICU care

**Comparison:** Control (ICU care)

| Outcomes | **Anticipated absolute effects^*^** (95% CI) | | Relative effect (95% CI) | № of participants  (studies) | Certainty of the evidence (GRADE) | Comments |
| --- | --- | --- | --- | --- | --- | --- |
|  | **Risk with control** | **Risk with acupuncture** |  |  |  |  |
| **Minor adverse events reported** | 5 per 1,000 | **30 per 1,000** (2 to 501) | **RR 5.69**  (0.34 to 95.60) | 674 (3 studies) | ⨁◯◯◯  Very low ^a, b, c^ | The evidence is very uncertain about the effect of acupuncture on minor adverse events in ICU patients. |
| **Adverse reactions** | 552 per 1,000 | **182 per 1,000** (121 to 276) | **RR 0.33**  (0.22 to 0.50) | 229  (3 studies) | ⨁⨁◯◯  Low ^a, c^ | The results indicate that adverse reactions happened less with acupuncture compared to routine ICU care and Sham acupoint catgut embedding. However the level of certainty in the evidence is low. |
| **Adverse events and adverse reactions** | 97 per 1,000 | **72 per 1,000** (26 to 198) | **RR 0.74**  (0.27 to 2.04) | 903  (6 studies) | ⨁◯◯◯  Very low ^a, b, c^ | The evidence is very uncertain about the effect of acupuncture on minor adverse events and adverse reactions in ICU patients. |
| **ICU stay** | The mean ICU days ranged from 3.5-22 Days | **MD 1.45 Days lower**  (1.94 lower to 0.97 lower) | **-** | 2190 (15 studies) | ⨁◯◯◯  Very low ^a, b^ | There is evidence that acupuncture is associated with reduced ICU stay in ICU patients. However, the certainty in the evidence is very low. |
| **28 days Mortality** | 339 per 1,000 | **239 per 1,000** (198 to 286) | **OR 0.61**  (0.48 to 0.78) | 1335 (10 RCTs) | ⨁⨁⨁◯ Moderate ^a^ | There is moderate evidence that acupuncture is associated with reduced 28 days mortality in ICU patients. |

The table describes the GRADE rating for certainty of evidence, where both minor AEs reported, 28-days mortality and mortality meta-analysis were ranked of certainty in the evidence.

Reasons for GRADE reduction:

a. high risk of bias due to: no randomization, no blinding or poor reporting.

b. I2 more than 50% (high heterogeneity)

c. small sample size

CI: confidence interval; RR: Risk ratio; OR: odds ratio, MD: mean difference.

*The risk in the intervention group (and its 95% confidence interval) is based on the assumed risk in the comparison group and the relative effect of the intervention (and its 95% CI).
